# Trends and correlates of cesarean section rates over two decades in Nepal

**DOI:** 10.1186/s12884-020-03453-2

**Published:** 2020-12-09

**Authors:** Aliza K. C. Bhandari, Bibha Dhungel, Mahbubur Rahman

**Affiliations:** grid.419588.9 0000 0001 0318 6320 St. Luke’s International University Graduate School of Public Health, Tokyo, Japan

**Keywords:** Cesarean section, Trends, Correlates, Nepal, Operative delivery

## Abstract

**Background:**

Cesarean section (CS) is a major component of emergency obstetric care. There has been a substantial rise in the rate of CS in private institutions in Nepal which might reflect the successful implementation of delivery schemes introduced by the government extended to the private organizations alternatively, it may also reflect the need for more public health care facilities to provide maternal and child health care services. Hence, the objective of this study was to examine the trends in institutional-based CS rates in Nepal along with its correlates over time.

**Methods:**

We used the National Demographic and Health Survey (NDHS) data collected every 5 years, from 1996 to 2016. The trend in CS rates based on five waves of NDHS data along with its correlates were examined using multivariable logistic regression models after adjusting for socio-demographics and pregnancy-related variables.

**Results:**

We included 20,824 reproductive-aged women who had a history of delivery within the past 5 years. The population-based CS rate increased from 0.9% in 1996 [95% CI: (0.6–1.2) %] to 10.2% in 2016 [95% CI: (8.9–11.6) %, *p* < 0.01] whereas the institutional-based CS rate increased from 10.4% in 1996 [95% CI: (8.3–12.9) %] to 16.4% in 2016 [95% CI: (14.5–18.5) %, *p* < 0.01]. Private institutions had a nearly 3-fold increase in CS rate (8.9% in 1996 [95% CI: (4.8–16.0) %] vs. 26.3% in 2016[95% CI: (21.9–31.3) %]. This was also evident in the trend analysis where the odds of having CS was 3.58 times higher [95% CI: (1.83–7.00), *p <* 0.01] in 2016 than in 1996 in the private sectors, while there was no evidence of an increase in public hospitals (10.9% in 1996 to 12.9% in 2016; *p for trend* > 0.05). Education of women, residence, wealth index, parity and place of delivery were significantly associated with the CS rate.

**Conclusion:**

Nepal has observed a substantial increase in cesarean delivery over the 20 years, which might indicate a successful implementation of the safe motherhood program in addressing the Millennium Development Goals and Universal Health Care agenda on maternal and child health. However, the Nepal government should examine existing disparities in accessibility of emergency obstetric care services, such as differences in CS between public and private sectors, and promote equity in maternal and child health care services accessibility and utilization.

**Supplementary Information:**

The online version contains supplementary material available at 10.1186/s12884-020-03453-2.

## Background

Cesarean section (CS) is a major component of emergency obstetric care performed to reduce maternal and fetal morbidity and mortality [1]. However, some studies have shown the increased risks associated with cesarean section compared with normal vaginal delivery for both mother and her child, especially if the CS is not medically indicated [2]. According to World Health Organization’s (WHO) estimates, about 6.2 million unnecessary CS are being performed annually in the world [3]. WHO recommends the national cesarean section (CS) rate to be between 10 and 15% with < 10 and > 15% representing underuse and overuse of maternal and child health care services, respectively [3]. In 2015, WHO added that, population-based CS rates of more than 10% are not associated with a reduction in maternal and neonatal mortality rates; however, they did not include the association between stillbirths or maternal and perinatal morbidity and CS rates due to unavailability of data [4]. Furthermore, a cross-sectional ecological study conducted among all 194 WHO member states concluded that the population-level CS rates of up to approximately 19 per 100 live births were associated with lower maternal and neonatal mortality [5]. Thus, it is still not clear to what extent of CS rate is considered safe.

Despite this, there is an alarming increase in the CS rate worldwide, with a wide disparity between low and high-income countries [6]. In a recent study, which included data from 169 countries across the globe in 2015, the overall CS rate was reported to be 21.1%, twice as high as that estimated based on 2000 data [7]. A rate of more than 40% was observed in the Latin America and the Caribbean, followed by Northern America (32.3%), Oceania (31.1%), Europe (25%), Asia (20%) and Africa (7.3%) [7]. Another study based on the WHO Global Survey on Maternal and Perinatal Health conducted in 2007–2008 among nine Asian countries, observed the national CS rates between 14.7 and 46.2% [8]. With the rapid rise in the CS rate, there have been concerns over the growing disparity of obstetric care service access and utilization. The inequitable distribution of health care resources in certain regions with limited health care facilities has left millions of people without adequate access to such services and expose them to the hazards of unsafe delivery [8].

The determinants of CS are varied and complex [9]. Several studies indicated that the rise in CS rate may be associated with the socio-demographic influences [10], and natal and antenatal factors such as maternal age, socio-economic index (education, wealth index, occupation) [11], place of residence [12], maternal body mass index (BMI) [13], birth order and birth weight of the child [14], previous experience with CS [15], obstetric complications [16], maternal preference [17] and place of delivery [18]. The influence of these factors varied among different populations [19].

Nepal expanded the emergency obstetric care services by promoting Skilled Birth Attendant (SBA) training programs in almost all districts of the country, which was implemented successfully to meet the Millennium Development Goals (MDG) target of reducing the maternal mortality rate by three-quarters to 134 per 100,000 live births by 2015. In addition to SBA training, the safe delivery incentive program was introduced by the Nepal Government in 2005 to promote the institutional-based deliveries. This program provided a cash incentive of Nepalese rupee (NPR) 500, NPR1000, NPR1500 (1 United States Dollar = NPR118.7) for the residence of Terai, Hills and Himalayan regions respectively, to women who delivered their baby in an institution. The incentive was also provided to the service providers based on the number of deliveries attended in a health facility or even for home deliveries [20]. In 2009, with the introduction of the Aama Suraksha Program, the out-of-pocket payments associated with institutional-based deliveries in all government health care facilities as well as in some private medical college hospital facilities were abolished [21]. Similarly, women were provided with an additional incentive of NPR400 for completing four antenatal checkup visits and first post-natal checkup visit in addition to the institutional delivery since 2009/2010. All these factors may have played important roles in reducing maternal and child health mortality rates by increasing CS services [22, 23]. However, most of these programs focused on the Basic Emergency Obstetric Care (BEmOC) Services only and incorporating the Comprehensive Emergency Obstetric Care (CEmOC) services in the hospitals was much slower indicated by the fact that more than a third of districts did not have CS capabilities in the public sector prior to 2010.

A study reported that the increase in CS rate in Nepal might be partly driven by the private sectors [24]. Large disparity in CS utilization in the urban and rural areas has also been reported [25]. Some socio-demographic factors such as age, education, economic stability and having an educated partner are associated with population-based CS rates [26, 27]. In a developing country like Nepal, where resources are not distributed evenly at hospitals and health facilities, analysis of the entire population does not give a clear picture of existing disparities in health care utilization. The rates of cesarean section in the health care facilities depend on the number of people they serve and clearly, the flow of patients in private facilities is more compared to government health care facilities [4]. Therefore, it would be inappropriate to apply the population-based cesarean rates at facility level as difference might exist in between private and government facilities. Many studies lacked a comparative trend analysis considering these differences between private and public sectors with regard to the determinants leading to increase in the CS rates. Similarly, the effects of various socio-demographic and natal and antenatal factors of women on the CS rate remain unexplored despite the fact that understanding these correlates might shed more light on existing disparities within the country. Also, in light of the significant rise in CS rates both globally and in Nepal there is a need to track whether this progress is inequitable or not. Thus, the objective of this study is to examine the trends in institutional-based CS in Nepal over two decades along with its correlates over time in Nepal.

## Methods

### Study design

For this study, the National Demographic and Health Survey (NDHS) data collected in Nepal in the years 1996, 2001, 2006, 2011 and 2016 were used with the permission obtained from the Demographic and Health Survey program department, USAID [28]. The NDHS collected data based on nationally representative samples by multi-stage cluster sampling design every 5 years in Nepal similar to that of many other developing countries using census ward as a primary sampling unit.

### Study participants

Overall, data on pregnancy and childbirth history was collected from 53,484 women aged 15 to 49 years that yielded an average response rate of 98.2%. This study only included women who responded to the NDHS question on whether their last birth in the last 5 years was through CS delivery. Women who had missing or erroneous records for the outcome of interest (cesarean section) were excluded from the analysis. In total, 20,824 women had a history of delivery in the last 5 years and information on whether CS was performed (3827 in 1996, 4730 in 2001, 4182 in 2006, 4079 in 2011 and 4006 in 2016).

### Outcome of interest

The main outcome of interest was cesarean section delivery which was measured in the National Demographic and Health Survey using a binary response “Yes” or “No”. Institutional-based CS rate is defined as the operative delivery performed in private or public health care facilities per 100 live births.

### Independent variables

#### Socio-demographics

This study included the following independent variables: women’s age, religion, education (less than or equal to primary education which is up to 5th grade of schooling versus more than or equal to secondary which is above 5th grade of schooling), residence, region (five developmental regions)/ province (seven provinces), age at first marriage and age at first birth, occupation, BMI, wealth index (divided into five equal quintiles from poorest to richest). In the NDHS, households are given scores based on the number and kinds of consumer goods they own, ranging from a television to a bicycle or car, and housing characteristics such as source of drinking water, toilet facilities, and flooring materials. These scores are derived using principal components analysis. National wealth quintiles are compiled by assigning the household score to each usual household member, ranking each person in the household population by her/his score, and then dividing the distribution into 12. Housing characteristics and household population have five equal categories, each comprising 20% of the population. Husband’s education was classified in a similar way as the women’s education. Provincial system was introduced in Nepal after 2015; thus, analysis based on 2016 data only included this variable. In 2015, one district was divided into two and two other districts were merged together to form a single district in Nepal. Hence, a separate analysis which included province as a covariate is shown in “Additional file 1”.

#### Natal and antenatal factors

We also included variables such as place of institutional delivery as “Government sector versus Non-Government sector”, number of antenatal care visits and size of the infant at birth in the analysis.

### Statistical analysis

We conducted univariate analysis and presented a weighted frequency. Bivariable analysis was conducted using the chi-square test or Fisher’s exact test as appropriate. Furthermore, bivariable logistic regression was performed and those covariates which were significant in the bivariable model at *p* < 0.05 were kept in the multivariable logistic regression model. Trend analysis was performed using logistic regression analysis after combining all the data across survey waves.

We conducted separate multivariable logistic regression models for each of the five waves of data. We excluded variables showing high collinearity, such as age at first marriage and husbands’ education, from all the regression models. Women’s BMI was also excluded due to high number of missing values across different waves of data. We also performed multivariable logistic regression analysis stratified by place of delivery (government versus private). We used *p* < 0.05 to indicate statistical significance in the bivariable and multivariable models. Weight variables included in the DHS databases considered the clustered nature of the data as well as other complex survey design issues. We used sample weight variable and conducted all the statistical analyses using “svy” command to account for clustering of the survey data.

Because of the very low number of events based on institutional births in the year 1996 and 2001, we did not report logistic regression analysis for these two survey waves and included them in “additional file 1” instead. Similarly, to examine the association with province variable (available only for the 2016 survey wave data), we fitted separate logistic regression model (“Additional file 1”). Stata IC version 15.1 was used for data coding and analysis.

### Ethical consideration

This study analyzed data extracted from the NDHS 1996, 2001, 2006, 2011 and 2016 surveys. The ethical clearance for these surveys was obtained from Nepal Research Council and ICF Macro Institutional Review Board in Calverton, Maryland, USA. The DHS data are publicly accessible while we obtained the permission to use it in May 2019 after DHS reviewed our proposal and we accepted the terms and conditions attached with data sharing policy [28]..

## Results

In total, 20,824 women aged 15–49 years had a history of childbirth within the last 5 years of interview and information on whether they had CS delivery. Sociodemographic characteristics of the women based on five waves of survey data are shown in Table 1 (all birth versus institutional birth). Of total participants, the majority of women were aged between 20 and 29 years and identified their religion as Hindu.

**Table 1:** Socio-demographic characteristics of all women of reproductive age grouped by survey waves

	NDHS-1996	Inst. births ***N*** = 334%	NDHS-2001	Inst. births ***N*** = 235%	NDHS-2006	Inst. births ***N*** = 854%	NDHS-2011	Inst. births ***N*** = 1682%	NDHS-2016	Inst. births ***N*** = 2485%
	All births ***N*** = 3821%		All births ***N*** = 4693%		All births ***N*** = 4182%		All births ***N*** = 4079%		All births ***N*** = 4006%	
Age of women										
≤ 19	10.6	10.9	8.0	10.5	8.0	11.5	8.0	11.1	8.4	9.8
20–29	58.7	71.7	57.4	61.4	63.6	65.9	36.6	67.6	66.3	68.0
30 +	30.7	17.4	34.6	28.1	28.4	22.6	28.4	21.3	25.3	22.2
Religion										
Hindu	85.9	88.0	84.1	88.4	84.9	85.1	83.0	86.0	85.6	86.0
Others	14.1	12.0	15.9	11.6	15.1	14.9	17.0	14.0	14.4	14.0
Education										
No education	79.2	41.8	72.7	48.8	57.9	28.1	43.9	24.2	31.5	20.3
≤ Primary	11.4	16.3	14.3	12.4	18.3	17.9	20.1	18.2	19.4	16.8
≥ Secondary	9.4	41.9	12.9	38.8	23.8	54.0	36.0	57.6	49.1	62.9
Residence										
Rural	93.5	64.4	93.2	83.9	86.8	66.9	89.9	81.1	44.4	35.1
Urban	6.5	35.6	6.8	16.1	13.2	33.1	10.1	18.9	55.6	64.9
Cesarean section										
No	99.1	89.6	99.6	92.4	96.7	84.3	94.8	87.0	89.8	83.6
Yes	0.9	10.4	0.4	7.6	3.3	15.7	5.2	13.0	10.2	16.4
Age at first marriage										
≤ 19	88.5	74.1	87.7	77.5	83.7	68.3	77.7	68.0	74.2	67.8
20–29	11.3	25.0	12.2	22.5	16.0	31.3	21.9	31.6	25.0	31.0
30 +	0.2	0.9	0.1	0	0.3	0.4	0.4	0.4	0.8	1.2
Age at first birth										
≤ 19	61.7	46.4	60.3	51.5	58.2	45.6	52.8	43.9	50.7	44.9
20–29	37.5	50.2	39.0	47.3	40.6	51.7	46.0	54.4	48.0	53.3
30 +	0.8	3.4	0.7	1.2	1.2	2.7	1.2	1.7	1.3	1.8
Occupation										
Not working	16.9	41.4	16.2	26.1	19.4	36.2	27.7	40.2	38.7	41.8
Working	83.1	58.6	83.8	73.9	80.6	63.8	72.3	59.8	61.3	58.2
Wealth index										
Poorest	14.3	22.1	15.3	18.2	23.5	8.4	23.6	8.9	20.5	13.0
Poorer	20.4	25.7	18.5	17.9	21.1	10.4	21.7	14.8	21.0	17.3
Middle	19.2	17.3	15.8	21.0	20.0	12.3	21.0	19.9	21.6	21.7
Richer	18.9	18.6	22.7	19.9	18.5	22.0	18.0	24.8	20.8	24.2
Richest	27.1	16.3	27.8	23.0	16.9	46.9	15.7	31.6	16.1	23.8
Husband’s education										
No education	38.8	13.3	34.9	20.3	23.5	12.0	21.0	10.8	13.5	8.2
≤ Primary	23.9	15.6	24.7	18.7	28.8	20.2	23.7	17.1	21.2	16.7
≥ Secondary	37.2	70.7	38.1	58.6	47.2	67.7	54.8	71.9	64.3	74.1
Missing	0.2	0.3	2.3	2.4	0.5	0.1	0.5	0.2	1.0	1.0
Body mass index										
High risk weight	27.5	25.3	28.6	27.4	30.6	33.1	14.8	16.2	18.1	19.0
Normal	70.8	73.1	71.3	71.9	68.7	66.3	33.8	31.5	32.4	31.3
Missing	1.7	1.6	0.2	6.2	0.7	0.6	51.4	52.3	49.5	49.7
Size of the baby										
Smaller than average	25.7	16.3	21.3	21.9	18.5	15.6	15.6	13.2	16.7	16.6
Average	42.9	48.3	55.3	54.3	58.1	60.4	65.6	66.4	67.1	65.8
Larger than average	31.4	35.4	23.4	23.8	23.4	24.0	18.8	20.4	16.3	17.6
Birth order										
First	21.7	39.9	20.8	37.6	26.9	49.4	31.4	48.1	37.5	48.8
Second +	78.3	60.1	79.2	62.4	73.0	50.6	68.6	51.9	62.5	51.2
Place of delivery										
Home	91.7	0	94.8	0	79.2	0	59.8	0	38.1	0
Government sector	6.2	75.1	2.0	38.1	14.4	69.2	28.3	70.4	45.6	73.6
Private sector	2.1	24.9	3.2	61.9	6.4	30.8	11.9	29.6	16.3	26.4
Antenatal care visits										
≤ 3	88.2	51.8	85.8	54.1	70.5	39.6	49.9	25.9	30.6	17.7
≥ 4	9.0	47.4	14.0	45.6	29.5	60.4	50.1	74.1	69.4	82.3
Missing	2.8	0.8	0.2	0.3						
Development Region										
Eastern	21.1	20.5	23.2	20.1	21.7	18.9	24.1	26.2	23.1	24.1
Central	33.2	48.5	32.3	35.7	32.7	46.4	31.1	31.4	35.4	33.4
Western	20.0	18.3	19.2	25.2	18.6	17.7	19.7	21.0	18.9	20.0
Mid-western	15.6	6.6	14.7	12.6	12.6	9.8	14.4	12.3	14.0	12.7
Far-western	10.1	6.1	10.6	6.4	14.4	7.2	10.6	9.1	8.6	9.8

*NDHS* National Demographic and Health Survey, *Inst. Births* Institutional-based deliveries

All births are total births, including home delivery and institutional deliveries in the respective survey rounds, whereas institutional births are the total number of births in an institution in the respective survey waves

Overall, the frequency of women with secondary education or above increased from 9.4% in 1996 to nearly 50% in 2016. The level of education was higher throughout the survey waves among women who had an institutional birth. Until 2011, the majority of women were residing in rural areas. However, in 2016, two-thirds of the women who gave birth in an institution were from urban residences (64.9%). About 60% of women who had an institutional delivery were employed, and more than 70% belonged to average or higher wealth quintiles in 2016, and almost 51% had their first child at the age of ≤ 19. The majority of women had normal BMI, and education level of their husband increased over time.

Until 2016, the majority of births took place at home, with just 0.9% of births occurring in a facility in 1996; this increased to 18% in 2006 and 62% in 2016. Most of the respondents were multiparous (78.3% in 1996 and 62.5% in 2016). However, there was a significant shift in primiparous women from 22% in 1996 to 37.5% in 2016. Majority of women had an average-sized infant throughout the survey years (nearly 43% in 1996 to 65.8% in 2016). The rate of complete four antenatal care visits or more, as recommended by WHO, increased about 8 -fold from 1996 to 2016 with a higher rate among women who had institutional deliveries. Eastern, Central and Western developmental regions together comprised of more than 70% of the study participants in all survey waves. Similarly, with the addition of the provincial system in 2016, province 2 had the highest number of participants (24.1%).

**Fig. 1:**
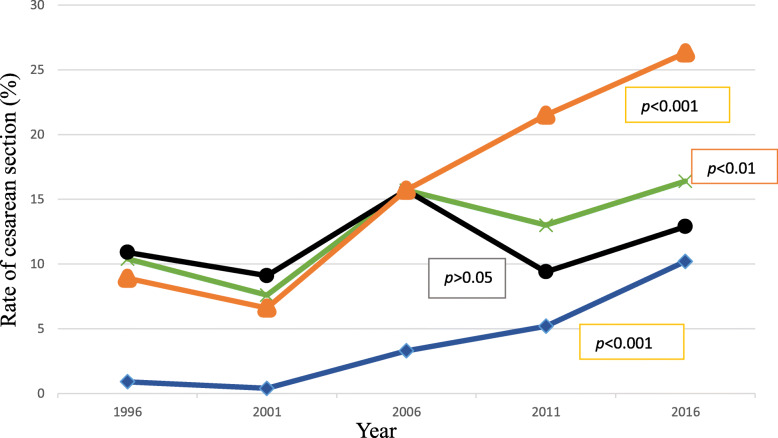
Trend analysis of cesarean section deliveries in Nepal, 1996–2016. Data are the rate of cesarean section deliveries in each NDHS survey waves where blue line with close diamonds represents rate of population-based CS deliveries, green line with cross indicates rate of institutional-based CS deliveries, orange line with close triangles indicates the rate of CS deliveries in private sectors and black line with close circles indicates the rate of CS deliveries in public sectors. *p-value* was obtained from logistic regression analysis with the year as a linear term and adjusted for all covariates under analysis

The population-based CS rate had increased 10-fold, from 0.9% in 1996 [95% CI: (0.6–1.2) %] to 10.2% in 2016 [95% CI: (8.9–11.6) %, *p* < 0.01] whereas the institutional-based CS rate increased from 10.4% in 1996 [95% CI: (8.3–12.9) %] to 16.4% in 2016 [95% CI: (14.5–18.5) %, *p* < 0.01]. Private institutions had a nearly 3-fold increase in CS rate (8.9% in 1996 [95% CI: (4.8–16.0) %] vs. 26.3% in 2016[95% CI: (21.9–31.3) %]. This was also evident in the trend analysis where the odds of having CS was almost 4-fold higher [OR: 3.58, 95 95% CI: (1.83–7.00), *p* < 0.01] in 2016 than in 1996 in the private sectors while there was no evidence of an increase in public hospitals (10.9% in 1996 to 12.9% in 2016; *p for trend* > 0.05). (Fig. 1 and Table 2).

**Table 2:** Trend of institutional-based cesarean section stratified by the place of delivery based on bivariable and multivariable logistic regression analysis

	Public sector		Private sector	
	cOR (CI)	aOR (CI)	cOR (CI)	aOR (CI)
Year				
1996	1	1	1	1
2001	0.82 (0.48–1.40)	0.87 (0.50–1.51)	0.71 (0.36–1.42)	0.93 (0.47–1.83)
2006	1.53 (0.95–2.45)	1.11 (0.71–1.75)	1.89 (0.93–3.86)	1.94 (0.95–3.94)
2011	0.85 (0.56–1.28)	0.84 (0.54–1.29)	2.78 (1.43–5.39) **	2.56 (1.31–4.98) **
2016	1.21 (0.84–1.74)	1.22 (0.82–1.80)	3.63 (1.85–7.11) ***	3.58 (1.83–7.00) ***

Number of cases included in the multivariable model *N* = 5090

*cOR* crude odds ratio obtained from bivariable analysis

*aOR* adjusted odds ratio (adjusted for all covariates under analysis for each survey wave)

*CI* 95% confidence interval

** = *P* < 0.01, *** = *P* < 0.001

Caesarean section rates varied across most socio-demographic and pregnancy-related variables, at each time point/survey (Table 3). Multivariable logistic regression analyses showed that women who had their baby after 30 years of age were nearly five times more likely to have CS in all of the survey waves than the women who had early childbirth and this association was highly significant in 2011 and 2016 survey waves than in 2006. Women had higher likelihood of CS for their first child in 2006, but similar results were not observed in recent survey years. With the increase in wealth index, the likelihood of having a cesarean section increased nearly 4-fold in 2016. However, it was not statistically significant in other survey waves. Women who delivered larger sized infants had a higher likelihood of getting CS than their counterparts almost in all survey waves (Table 4).

**Table 3:** Proportion of institutional-based cesarean section and its association with selected covariates in respective survey waves

	Cesarean section (weighted %)				
	1996 ***N*** = 334	2001 ***N*** = 235	2006 ***N*** = 854	2011 ***N*** = 1682	2016 ***N*** = 2485
Age of women					
≤ 19	4 (13.0)	1 (5.9)	14 (16.0)	10 (6.0)	24 (9.5)
20–29	25 (10.1)	12 (4.4)	71 (16.1)	143 (12.3)	243 (15.8)
≥ 30	5 (10.1)	6 (8.5)	26 (14.4)	61 (18.6)	99 (21.4)
*p-value*	0.729	0.766	0.896	< 0.01	< 0.01
Religion					
Hindus	31 (11.1)	16 (7.2)	97 (14.9)	189 (13.2)	311 (16.2)
Non-Hindus	3 (5.9)	3 (10.2)	14 (20.1)	25 (11.8)	55 (17.6)
*p-value*	0.189	< 0.05	0.489	0.697	0.642
Education					
No education	15 (13.1)	9 (7.8)	21 (13.8)	28 (8.8)	52 (12.1)
Primary or less	4 (5.9)	1 (2.7)	20 (16.2)	35 (13.3)	46 (11.3)
Secondary +	15 (9.4)	9 (8.8)	70 (16.5)	151 (14.6)	268 (19.2)
*p-value*	0.050	0.307	0.750	0.069	< 0.001
Residence					
Rural	18 (9.6)	8 (4.5)	56 (14.0)	100 (10.9)	106 (13.7)
Urban	16 (11.8)	11 (23.7)	55 (19.1)	114 (22.0)	260 (17.9)
*p-value*	0.391	< 0.001	0.148	**<** 0.001	< 0.05
Age at first birth					
≤ 19	13 (8.8)	5 (4.1)	38 (11.0)	64 (8.7)	119 (10.7)
20–29	17 (9.8)	13 (10.2)	65 (18.4)	139 (15.7)	228 (20.2)
≥ 30	4 (42.6)	1 (53.6)	8 (42.8)	11 (35.8)	19 (45.2)
*p-value*	< 0.01	< 0.001	< 0.01	< 0.001	< 0.001
Birth order					
Second +	13 (7.1)	9 (5.2)	39 (10.5)	107 (12.2)	164 (14.2)
First	21 (15.3)	10 (11.4)	72 (21.0)	107 (13.8)	202 (18.7)
*p-value*	< 0.01	< 0.05	< 0.01	0.382	< 0.05
Occupation					
Not working	15 (8.7)	7 (10.0)	60 (24.0)	102 (16.5)	164 (18.4)
Working	19 (11.7)	12 (0.67)	51 (11.0)	112 (10.6)	202 (14.9)
*p-value*	0.098	0.121	< 0.001	< 0.01	0.061
Wealth index					
Poorest	7 (10.2)	6 (10.8)	6 (10.6)	12 (9.2)	27 (7.4)
Poorer	10 (13.1)	4 (9.5)	6 (7.3)	9 (2.8)	40 (9.2)
Middle	4 (6.3)	3 (4.2)	8 (9.3)	34 (13.6)	63 (12.6)
Richer	6 (11.5)	3 (7.7)	17 (10.0)	48 (13.0)	89 (14.0)
Richest	7 (9.5)	3 (6.4)	74 (22.8)	111 (18.4)	147 (32.4)
*p-value*	0.453	0.176	< 0.001	< 0.001	< 0.001
Size of the baby					
Average	12 (17.0)	10 (6.1)	56 (11.7)	136 (11.8)	224 (15.0)
Large	19 (6.9)	4 (6.8)	35 (23.2)	49 (16.3)	75 (21.4)
Small	3 (6.4)	5 (11.1)	19 (18.9)	28 (12.9)	67 (16.8)
*p-value*	< 0.001	0.243	< 0.05	0.305	< 0.05
Place of delivery					
Government	27 (10.9)	10 (9.1)	71 (15.7)	112 (9.4)	138 (12.9)
Private/others	7 (8.9)	9 (6.6)	40 (15.7)	102 (21.5)	228 (26.3)
*p-value*	0.558	< 0.05	0.992	< 0.001	< 0.001
ANC Visits					
Less than 4	15 (8.5)	127 (4.6)	29 (11.0)	32 (9.6)	44 (12.0)
4 or more	19 (12.7)	107 (10.4)	82 (18.8)	182 (14.1)	322 (17.4)
*p-value*	0.154	< 0.05	< 0.05	0.081	< 0.05
Region					
Far western	1 (2.2)	2 (7.3)	8 (7.6)	18 (6.3)	23 (5.5)
Eastern	10 (17.6)	4 (8.4)	21 (13.4)	61 (16.1)	105 (19.1)
Central	14 (8.2)	13 (15.1)	40 (17.9)	15 (16.7)	96 (19.4)
Western	5 (9.2)	0 (0)	30 (18.3)	43 (9.5)	110 (20.2)
Mid-western	4 (11.1)	0 (0)	12 (11.1)	27 (7.5)	32 (5.8)
*p-value*	< 0.05	< 0.01	0.204	< 0.001	< 0.001

*P* value obtained from chi square or Fisher’s exact test analysis

**Table 4:** Bivariable and multivariable logistic regression analysis for the association between institutional cesarean section and selected covariates in survey waves 2006, 2011 and 2016

	2006		2011		2016	
	cOR (CI)	aOR (CI)	cOR (CI)	aOR (CI)	cOR (CI)	aOR (CI)
Region (Ref: Far-western)						
Eastern	1.88 (0.85–4.19)	1.31 (0.57–3.03)	2.84 (1.41–5.73) **	1.99 (0.93–4.25)	4.04 (2.35–6.95) ***	3.03 (1.77–5.19) ***
Central	2.67 (1.42–5.00) **	1.54 (0.78–3.02)	2.98 (1.53–5.78) **	2.15 (1.05–4.40) *	4.14 (2.40–7.13) ***	2.58 (1.52–4.40) **
Western	2.73 (1.35–5.51) **	1.91 (0.91–4.00)	1.56 (0.76–3.18)	1.38 (0.65–2.90)	4.34 (2.57–7.35) ***	2.91 (1.69–4.99) ***
Mid-western	1.52 (0.82–2.81)	1.26 (0.58–2.73)	1.21 (0.54–2.67)	1.19 (0.54–2.64)	1.05 (0.53–2.07)	1.07 (0.54–2.12)
Age at first birth (Ref: ≤ 19)						
20–29	1.82 (1.12–2.93) *	1.71 (1.01–2.90) *	1.95 (1.36–2.81) ***	1.62 (1.08–2.43) *	2.12 (1.58–2.84) ***	1.49 (1.09–2.02) *
30+	6.04 (1.94–18.8) **	4.60 (1.70–12.43) **	5.85 (2.18–15.6) ***	4.99 (1.62–15.38) **	6.89 (3.36–14.13) ***	4.85 (2.27–10.39) ***
Birth order (Ref: Second +)						
First	2.26 (1.26–4.07) **	2.01 (1.10–3.68) *	1.15 (0.84–1.58)	0.99 (0.69–1.43)	1.40 (1.05–1.86) *	1.21 (0.88–1.67)
Child size (Ref: Average)						
Large	2.28 (1.12–4.64) *	2.49 (1.41–4.39) **	1.46 (0.90–2.35)	1.59 (0.95–2.67)	1.54 (1.14–2.08) **	1.62 (1.22–2.14) **
Small	1.75 (0.96–3.21)	2.05 (1.02–4.12) *	1.11 (0.59–2.06)	1.17 (0.62–2.20)	1.15 (0.83–1.58)	1.36 (0.97–1.90)
ANC visit (Ref: 3 or less)						
4 and above	1.88 (1.07–3.31) *	1.40 (0.71–2.75)	1.55 (0.94–2.55)	1.35 (0.75–2.45)	1.54 (1.03–2.28) *	1.28 (0.87–1.87)
Occupation (Ref: Not working)						
Working	0.39 (0.24–0.62) ***	0.50 (0.29–0.88) *	0.60 (0.43–0.83) **	0.81 (0.57–1.17)	0.78 (0.60–1.01)	1.11 (0.83–1.49)
Wealth index (Ref: Poorest)						
Poorer	0.66 (0.24–1.79)	0.65 (0.24–1.77)	0.29 (0.08–0.99) *	0.28 (0.09–0.91) *	1.28 (0.69–2.36)	0.95 (0.51–1.80)
Middle	0.86 (0.30–2.43)	0.70 (0.24–2.09)	1.56 (0.61–3.97)	1.24 (0.50–3.07)	1.80 (0.98–3.31)	1.28 (0.69–2.36)
Richer	0.93 (0.36–2.37)	0.68 (0.24–1.91)	1.49 (0.59–3.74)	0.90 (0.33–2.43)	2.04 (1.21–3.45) **	1.33 (0.77–2.28)
Richest	2.48 (1.17–5.29) *	1.77 (0.66–4.79)	2.23 (0.90–5.53)	0.98 (0.35–2.78)	6.01 (3.57–10.11) ***	3.85 (2.12–6.98) ***
Education (Ref: No education)						
Primary or less	1.21 (0.66–2.23)	0.92 (0.47–1.80)	1.59 (0.85–2.97)	1.56 (0.80–3.06)	0.93 (0.59–1.47)	0.88 (0.54–1.44)
Secondary +	1.24 (0.62–2.46)	0.61 (0.33–1.10)	1.77 (1.06–2.96) *	1.31 (0.66–2.57)	1.73 (1.18–2.53) **	0.94 (0.60–1.47)
Residence (Ref: Rural)						
Urban	1.44 (0.87–2.38)	1.10 (0.63–1.90)	2.31 (1.63–3.26) ***	1.95 (1.34–2.83) ***	1.36 (1.01–1.85) *	0.83 (0.61–1.14)
Place of delivery (Ref: Government sector)						
Private sector	1.00 (0.55–1.81)	1.33 (0.78–2.26)	2.63 (1.87–3.70) ***	2.37 (1.63–3.44) ***	2.42 (1.76–3.32) ***	2.22 (1.62–3.03) ***

Number of cases included in the multivariable model *N* = 5011 (10 women did not report on ANC visits)

*OR* adjusted odds ratio (adjusted for all covariates under analysis for each survey round)

*CI* 95% confidence interval

* = *P* < 0.05, ** = *P* < 0.01, *** = *P* < 0.001

All regions had a higher CS rate than the far western development region in all survey waves. In 2016, women residing in the Eastern, Western and Central regions had two- to three-fold likelihood of having CS than women residing in the far western region. In addition, women in urban areas had a nearly two-fold likelihood of getting CS than those residing in the rural areas based on 2011 survey data only. The likelihood of getting CS was more than two-fold higher in private sectors than in government sectors in the last two survey rounds. Separate multivariable logistic regression analysis stratified by the place of delivery showed, by and large, similar correlates (Table 5). In comparison with the other five provinces, province three and four had nearly 2.5 times higher risk of having CS (Additional file 1).

**Table 5:** Multivariable logistic regression analysis for the association between institutional cesarean section and selected covariates stratified by place of delivery in survey waves 2006, 2011 and 2016

	Public, OR (CI)	2006	Public, OR (CI)	2011	Public, OR (CI)	2016
		Private, OR (CI)		Private, OR (CI)		Private, OR (CI)
**Region (Ref:** 5)						
Eastern	1.55 (0.32–7.53)	0.57 (0.14–2.37)	1.43 (0.54–3.82)	5.08 (1.06–24.34) *	2.05 (1.13–3.73) *	54.44 (5.67–522.4) **
Central	1.64 (0.36–7.41	1.09 (0.43–2.76)	1.28 (0.54–3.01)	6.83 (1.42–32.81) *	1.67 (0.92–3.03)	52.22 (5.33–511.9) **
Western	2.16 (0.43–10.77)	1.12 (0.40–3.14)	1.04 (0.45–2.43)	2.78 (0.55–13.92)	2.67 (1.51–4.72) **	30.35 (3.11–296.0) **
Mid-western	0.50 (0.10–2.58)	2.97 (1.17–7.49) *	0.84 (0.32–2.18)	2.86 (0.59–13.87)	1.00 (0.49–2.00)	3.04 (0.20–45.2)
**Age at first birth (Ref:** ≤ 19**)**						
20–29	1.74 (0.97–3.12)	2.13 (1.12–4.05) *	1.61 (0.90–2.88)	1.49 (0.78–2.83)	1.42 (0.99–2.05)	1.55 (0.84–2.89)
30+	4.11 (1.42–11.93) *	32.48 (2.70–391.1) **	4.86 (0.82–28.91)	6.64 (1.28–34.36) *	4.48 (1.88–10.65) **	11.96 (0.38–42.3) ***
**Birth order (Ref:** Second +**)**						
First	2.30 (1.03–5.13) *	1.34 (0.61–2.93)	0.70 (0.42–1.17)	1.65 (0.96–2.86)	1.17 (0.82–1.69)	1.27 (0.75–2.2)
**Child size (Ref:** Average**)**						
Large	2.78 (1.36–5.67) **	2.53 (1.18–5.43) *	1.07 (0.57–1.99)	2.76 (1.28–5.97) **	1.52 (1.03–2.22) *	2.06 (1.10–3.83) *
Small	1.88 (0.74–4.77)	2.24 (0.70–7.15)	0.58 (0.23–1.46)	2.70 (1.16–6.27) *	1.52 (0.99–2.32)	1.17 (0.63–2.18)
**ANC visit (Ref:** 3 or less)						
4 and above	1.22 (0.56–2.67)	1.44 (0.65–3.20)	1.18 (0.63–2.19)	1.76 (0.59–5.29)	1.15 (0.63–2.09)	1.46 (0.80–2.67)
**Occupation (Ref:** Not working**)**						
Working	0.47 (0.24–0.91) *	0.47 (0.21–1.08)	0.87 (0.50–1.51)	0.73 (0.40–1.34)	0.93 (0.66–1.32)	1.58 (0.99–2.51)
**Wealth index (Ref:** Poorest**)**						
Poorer	0.41 (0.12–1.34)	0.70 (0.14–3.35)	0.22 (0.05–0.96) *	0.48 (0.10–2.27)	1.21 (0.59–2.48)	0.45 (0.13–1.58)
Middle	0.32 (0.09–1.16)	1.06 (0.11–10.21)	0.92 (0.27–3.09)	1.68 (0.43–6.46)	1.28 (0.65–2.52)	0.94 (0.31–2.84)
Richer	0.75 (0.28–1.99)	0.09 (0.02–0.36) **	0.68 (0.21–2.20)	1.14 (0.29–4.49)	1.54 (0.77–3.08)	0.84 (0.31–2.23)
Richest	1.03 (0.33–3.23)	1.63 (0.45–5.88)	1.00 (0.27–3.74)	0.97 (0.24–3.83)	3.91 (1.90–8.09) ***	2.82 (1.05–7.56) *
**Education (Ref:** No education**)**						
Primary or less	0.59 (0.29–1.17)	3.95 (0.63–24.68)	1.64 (0.66–4.08)	1.45 (0.52–4.03)	1.29 (0.62–2.67)	0.50 (0.26–0.94) *
Secondary +	0.43 (0.26–0.73) **	2.22 (0.32–15.35)	1.32 (0.49–3.54)	1.33 (0.52–3.40)	1.13 (0.59–2.17)	0.82 (0.43–1.56)
**Residence (Ref:** Rural**)**						
Urban	0.93 (0.49–1.74)	1.35 (0.67–2.72)	0.56 (0.34–0.92) *	0.43 (0.23–0.79)**	1.14 (0.77–1.68)	1.28 (0.77–2.14)

Number of cases included in the multivariable model *N* = 5011 (10 women did not report on ANC visits)

*OR* adjusted odds ratio (adjusted for all covariates under analysis for each survey round)

*CI* 95% confidence interval

* = *P* < 0.05, ** = *P* < 0.01, *** = *P* < 0.001

## Discussion

This study highlighted the trends in institutional-based CS among Nepalese women over the past two decades using five survey waves (1996, 2001, 2006, 2011 and 2016) and also identified maternal socio-demographic and pregnancy-related factors associated with this trend.

Nepal observed a 6-percentage point rise in the institutional-based CS rate in 2016 compared to 1996 (10.4% versus 16.4%). However, the population-based CS rates have not exceeded the upper limit based on WHO guidelines [4]. This sharp increase in the rate of institutional-based CS rates in Nepal may have reflected the improved maternal and child health care access throughout the country. Compared to 1996 data, Nepal was able to significantly reduce maternal mortality rate (MMR) related to obstetric complications to meet the MDG 2015 of 50% MMR reduction using a very concrete National action plan and SBA training programs [29]. Similar escalating trend in the CS rates has been observed in other countries of the world. Western countries such as Germany, Italy, Denmark and Australia had CS rates over 10% during the 1990s, which increased 2–4 fold a couple of decades later [30]. Similarly, some of the developing countries in Asia also showed an upward trend. For example, CS rate increased to 24.5% in 2011 from 14.3% in 2007 in Bangladesh [10, 14]. India also showed a similar trend like that of Nepal with an institutional-based CS rate of 21.8% in 2016 compared to 11% reported in 1988 [31]. Several factors such as maternal education, wealth index, and accessibility to emergency obstetric care, women’s preferences and health-related policy factors of the country may be responsible for an upward trend in CS rates in these countries [32]. The increasing rate of CS is an important indicator of advancing health care services, especially for low-income countries like Nepal.

Several factors were identified as correlates of CS rate in this study. Nepal has shown a drastic change in the distribution of women by residence. A recent study reported that women living in rural areas have no or less access to emergency obstetric care [33, 34]. The lower CS rate in rural areas compared to that of urban areas might be associated with several existing barriers to maternal and child health care in rural areas. Also, women with higher socio-economic status [35], higher maternal age, and woman who delivered larger than average-sized infants were more likely to have CS delivery, although the latter two categories might be associated with medical indications [36] which was not analyzed in this study. This study identified that there is a high disparity in the utilization of cesarean section facilities across different developmental regions of Nepal. A study showed that Far-western and Mid-western developmental regions are underprivileged with inadequate health care facilities and low GDP [37]. This might be responsible for the higher CS rate observed in this study among regions with more advanced maternal and child health care services compared to those residing in a far western development region. Based on these findings, shifting resources from over-utilized areas to the areas where it is needed may be indicated.

The current study also identified that there are certain changes in the strength of correlates of CS from 2006 to 2016. In mid-2000s, correlates like region, wealth index and place of delivery were not associated at all or had very weak association with the institutional-based CS. However, over time the association became stronger reflecting the increase in inequitable access in CS service care utilization among few sub-groups of the population. Certain sub-groups of the population had far higher CS rate with stronger association than others in recent survey waves compared to 2006. Additionally, there had been vast advancement in the health care services as evidenced by increase in number of health care facilities and successful implementation of various incentive programs of Nepal Government to promote the institutional delivery [20]. However, the fact that certain subgroups of the population remain underprivileged still remains the same. Hence, Nepal government should focus more on reducing these disparities in between the sub-groups so that everyone in need could utilize the CS services from either public or the private health care facilities.

Another key finding of this study is that majority of primigravid women had delivered through CS. This is also true for other regions of the world as there are several factors influencing the preferences of a woman to undergo CS which include fear of labor pain, having a previous CS or living in a middle-income country, and so on [14, 38, 39]. Furthermore, women who delivered larger than average-sized infants had a 2-fold risk of getting CS than those who delivered an average-sized infant. This might be associated with some absolute and relative medical indications of CS, as suggested by Mylonas et al. [36].

The current study also observed the changes in the health care service utilization by pregnant women over the period of time. The health care utilization shifted from mostly home deliveries in 1996 to institutional deliveries in 2016. Therefore, the rising CS rate might indicate the progress in the development of maternal and child health care services of the country [34]. In contrast, the rapid increase of CS rate in the private organizations might be profit-driven or indicative of better implementation of safe motherhood and SBA training programs. Additional studies might shed more light on the accurate reasons for this increase in private organizations.

This study had several strengths. This is the first study showing the trend of institutional-based CS over a period of two decades in Nepal with the most recent data available. Also, the study used five waves of NDHS datasets, which includes a nationally representative sample population of Nepal. Therefore, our results are highly generalizable to the Nepalese population. Although there are few studies that showed the association between maternal socio-demographic characteristics and the CS rates, those were based on smaller sample size and non-representative populations [27]. In contrast, the current study focused on natal, antenatal factors and health care utilization variables, in addition to socio-demographics that might influence the rate of CS in the country. This study also reflected the existing disparities within various developmental regions of Nepal which might be useful to policymakers.

This study also had several limitations. Very low CS rates observed in 1996 and 2001 did not allow us to examine the correlates of CS for those years. Also, the NDHS data of Nepal did not have information about the type of cesarean section (emergency or elective), previous history of CS and medical indications of CS, which could have allowed us to examine the causes of CS and whether the CS performed was medically appropriate. A more robust analysis of the appropriateness of CS could have been done using the Robson score. However, due to the lack of data on these variables, this study was not able to conclude whether the CS was clinically indicated or not. Despite the limitations, the study provided important evidence with regard to the increase in CS rate and socio-demographics as well as service utilization correlates of CS in Nepal.

## Conclusion

This study, based on five waves of population-representative data, showed that the CS rate had increased substantially during the last few decades which represents a success story of Nepal in meeting the MDG goal, as well as UHC agenda of reducing maternal mortality and morbidity by enabling greater access to emergency obstetric care services like CS. This upward trend was fueled by procedures performed at private institutions where this rate experienced nearly 3-fold increase during 1996–2016. On the other hand, an almost unchanged CS rate in government institutes during this period suggests that there might be a persisting need for strengthening the comprehensive obstetric care services in the public sectors. However, a reasonable number of CS being performed in private organizations might be suggesting a commensurate rise in the number of women accessing private providers because of the incorporation of safe motherhood programs and incentive schemes of the Nepal government to encourage institutional deliveries into the private sectors as well. Considerable disparity in CS rates by developmental regions implicates that, inadequate number of medical institutions, along with a lack of maternal and health care facilities, are still widely prevalent in some regions. Hence, it is critical time for the Nepal government to develop policies which could help balance these prevailing disparities in the utilization of cesarean section procedure so that all Nepalese women can have equal access to maternal and child health care services.

## Supplementary Information

**Additional file 1.** Proportion of institutional based cesarean section and its association with selected covariates and Multivariable logistic regression for the association between institutional cesarean section and measured characteristics by the survey year.

## Data Availability

The National Demographic and Health Survey data of Nepal can be obtained from the USAID’s official website [28].

## Abbreviations

CS: Cesarean section
MDG: Millennium Development Goals
SBA: Skilled Birth Attendants
NDHS: National demographic and health survey
MCH: Maternal and child health
USAID: United States Agency for International Development
WHO: World health organization
BMI: Body mass index
ANC: Antenatal care
GDP: Gross Domestic Product
aOR: Adjusted odds ratio
OR: Odds ratio
cOR: Crude odds ratio
CI: Confidence interval
NPR: Nepalese Rupee
BEmOC: Basic emergency obstetric care
CEmOC: Comprehensive emergency obstetric care
