# Supplementary material for: Trends and correlates of cesarean section rates over two decades in Nepal
Source: BMC Pregnancy Childbirth. 2020 Dec 9;20:763. doi: 10.1186/s12884-020-03453-2 (PMC7724849; doi:10.1186/s12884-020-03453-2)
Supplement: Supplementary file 1 — Additional file 1. Proportion of institutional based cesarean section and its association with selected covariates and Multivariable logistic regression for the association between institutional cesarean section and measured characteristics by the survey year. [file 12884_2020_3453_MOESM1_ESM.docx]

Additional file 1. Proportion of institutional based cesarean section and its association with selected covariates

|  | NDHS 2016 | | |
| --- | --- | --- | --- |
|  | **Institutional births**  **%** | **Cesarean section**  **%** | **aOR (CI)** |
| Region |  |  |  |
| Eastern | 24.1 | 5.5 | 2.89(1.68-4.96)*** |
| Central | 33.4 | 19.1 | 1.29(0.67-2.51) |
| Western | 20.0 | 19.4 | 1.85(1.02-3.36)* |
| Mid-western | 12.7 | 20.2 | 1.08(0.55-2.13) |
| Far western | 9.8 | 5.8 | Ref |
| Age at first birth |  |  |  |
| ≤ 19 | 44.9 | 10.7 | Ref |
| 20-29 | 53.3 | 20.2 | 1.39(1.01-1.90)* |
| 30+ | 1.8 | 45.2 | 4.49(2.14-9.41)*** |
| Birth order |  |  |  |
| First | 48.8 | 14.2 | 1.22(0.89-1.68) |
| Second | 51.2 | 18.7 | Ref |
| Child size |  |  |  |
| Average | 65.8 | 15.0 | Ref |
| Large | 17.6 | 21.4 | 1.59(1.19-2.11)** |
| Small | 16.6 | 16.8 | 1.41(1.00-1.99)* |
| ANC visit |  |  |  |
| 3 or less | 17.7 | 12.0 | Ref |
| 4 and above | 82.3 | 17.4 | 1.20(0.82-1.74) |
| Occupation |  |  |  |
| Not working | 41.8 | 18.4 | Ref |
| Working | 58.2 | 14.9 | 1.00(0.73-1.36) |
| Wealth index |  |  |  |
| Poorest | 13.0 | 7.4 | Ref |
| Poorer | 17.3 | 9.2 | 1.05(0.56-1.98) |
| Middle | 21.7 | 12.6 | 1.54(0.83-2.86) |
| Richer | 24.2 | 14.0 | 1.46(0.84-2.53) |
| Richest | 23.8 | 32.4 | 4.40(2.40-8.07)*** |
| Education |  |  |  |
| No education | 20.3 | 12.1 | Ref |
| Primary or less | 16.8 | 11.3 | 0.80(0.49-1.28) |
| Secondary + | 62.9 | 19.2 | 0.80(0.52-1.25) |
| Residence |  |  |  |
| Rural | 35.1 | 13.7 | Ref |
| Urban | 64.9 | 17.9 | 0.77(0.56-1.06) |
| Place of delivery |  |  |  |
| Government sector | 73.6 | 12.9 | Ref |
| Private sector | 26.4 | 26.3 | 2.42(1.78-3.27)*** |
| Province |  |  |  |
| 1 | 18.5 | 20.5 | Ref (province 1, 2, 5, 6 and 7) |
| 2 | 18.4 | 11.7 | [2.49(1.60-3.86)***] |
| 3 | 20.6 | 24.9 |  |
| 4 | 9.6 | 25.1 |  |
| 5 | 18.8 | 11.2 |  |
| 6 | 4.4 | 6.3 |  |
| 7 | 9.8 | 5.5 |  |

NDHS= national demographic and health survey, aOR = adjusted odds ratio (adjusted for all the covariates under analysis), CI = confidence interval, [] = odds ratio and CI for province 3 and 4 compared with other provinces

Additional file 1. : Multivariate logistic regression for the association between institutional cesarean section and measured characteristics by the survey year.

|  | **1996, N = 334** | **2001, N = 159** |
| --- | --- | --- |
|  | **aOR**  **(CI)** | **aOR**  **(CI)** |
| **Region (Ref: Far-western)** |  |  |
| Eastern | 6.20 (3.87-9.92)*** | 0.79 (0.44-1.40) |
| Central | 3.36 (1.79-6.32)*** | 1.52 (0.98-2.46) |
| Western | 3.25 (1.24-8.49)* | - |
| Mid-western | 4.27 (2.58-7.07)*** | - |
| **Age at first birth (Ref:** ≤ 19**)** |  |  |
| 20-29 | 0.91 (0.48-1.73) | 2.24 (1.65-3.04)*** |
| 30+ | 6.48 (0.99-42.28) | - |
| **Birth order (Ref:** Second +**)** |  |  |
| First | 2.37 (1.27-5.26)** | 1.56 (0.70-3.50) |
| **Child size (Ref:** Average**)** |  |  |
| Large | 2.89 (1.58-5.26)** | 1.42 (0.58-3.46) |
| Small | 1.29 (0.54-3.04) | 1.67 (0.56-5.01) |

Number of cases missing in the model, N = 86

aOR: adjusted odds ratio (adjusted for covariates under analysis for each survey round based on number of events)

CI = confidence interval

**P* < 0.05, ***P* < 0.01, ****P* < 0.001

“-” = Not analyzed in the multivariate logistic regression model because of the low number of events (cesarean section)
